# Mechanisms of Exogenous Brassinosteroids and Abscisic Acid in Regulating Maize Cold Stress Tolerance

**DOI:** 10.3390/ijms26073326

**Published:** 2025-04-02

**Authors:** Tao Yang, Zelong Zhuang, Jianwen Bian, Zhenping Ren, Wanling Ta, Yunling Peng

**Affiliations:** 1 College of Agronomy, Gansu Agricultural University, Lanzhou 730070, China; yangtao990805@163.com (T.Y.); zhuangzl3314@gmail.com (Z.Z.); bjwen1018@163.com (J.B.); renzp1003@163.com (Z.R.); kellytwl@163.com (W.T.); 2 Gansu Provincial Key Laboratory of Aridland Crop Science, Gansu Agricultural University, Lanzhou 730070, China; 3 Gansu Key Laboratory of Crop Improvement & Germplasm Enhancement, Gansu Agricultural University, Lanzhou 730070, China

**Keywords:** maize (*Zea mays* L.), cold stress, hormone interactions, abscisic acid, brassinosteroid

## Abstract

Exogenous abscisic acid (ABA) and brassinosteroid (BR) play important roles in alleviating cold stress in maize. In this study, two maize inbred lines with differing cold tolerance were treated with exogenous ABA, BR, and their combined solution under cold stress conditions at 10 °C to investigate the effects of these treatments on the physiological characteristics of maize seedlings. The results indicated that cold stress significantly inhibited the growth of maize seedlings. Exogenous hormone treatments enhanced antioxidant enzyme activities and promoted the synthesis of osmolytes, thereby alleviating cold stress; however, the combined treatment (AR) did not significantly improve maize cold tolerance. Transcriptomic analysis revealed that pathways including plant hormone signal transduction, fatty acid elongation, and phenylpropanoid biosynthesis were involved in the interaction between ABA and BR. Weighted gene co-expression network analysis (WGCNA) identified four key candidate genes responsive to exogenous ABA and BR under cold stress, namely Zm00001eb343270, Zm00001eb401890, Zm00001eb206790, and Zm00001eb199820. Based on the gene annotation results, we speculate that ubiquitin-conjugating enzyme E2 O, tubulin–tyrosine ligase-like protein 12, the negative regulator of systemic acquired resistance SNI1, and mRNA stability regulators in response to DNA damage may be involved in regulating maize cold tolerance. These findings provide further evidence for the regulatory mechanisms by which exogenous ABA and BR affect maize cold tolerance and elucidate their interaction under cold stress.

## 1. Introduction

Maize (*Zea mays* L.), as a globally important cereal crop, plays a pivotal role in the production of food, feed, bioenergy, and industrial raw materials, thereby ensuring food security [1]. Originally from tropical regions, this crop is a warm-loving, short-day C4 plant that exhibits high sensitivity to low temperatures, with cold stress significantly affecting its growth and developmental processes [2]. Low temperature typically refers to a range of 0–15 °C and is one of the primary abiotic stress factors limiting plant growth and geographic distribution [3]. When ambient temperatures fall below 15 °C, maize growth is significantly inhibited, manifesting as impaired seed germination and symptoms in seedlings such as stunted growth, chlorosis, wilting, or even death [4]. During the seedling stage, cold stress not only markedly reduces the photosynthetic efficiency of maize leaves but also increases electrolyte leakage, resulting in significant declines in both fresh and dry biomass, with severe cases exhibiting water-soaked wilting symptoms [5]. As climate change leads to increasingly unstable weather patterns, enhancing maize tolerance to cold stress has become a key objective in global agricultural research.

Brassinosteroids (BRs) are an important class of steroid hormones in plants that are extensively involved in plant growth, development, and stress responses [6]. Studies have shown that BRs enhance plant stress tolerance by modulating photosynthesis, augmenting antioxidant capacity, and promoting osmotic adjustment under conditions of drought, salinity, and low-temperature stress [7,8,9]. For example, Sun et al. reported that exogenous application of BRs significantly improved the photosynthetic performance of maize under drought stress, enhanced antioxidant activity, and increased drought tolerance by modulating *ZmMYB* gene expression [10]. Mu et al. demonstrated that exogenous BRs can enhance the antioxidant capacity of rice seedlings, scavenge reactive oxygen species, and alleviate oxidative damage, thereby improving photosynthesis and salt tolerance [11]. Furthermore, Wang et al. indicated that exogenous brassinolide (BL) significantly mitigated the growth inhibition of rice seedlings under cold water stress, effectively enhancing germination rates and biomass while increasing the activities of antioxidant enzymes such as SOD and POD. Additionally, rice cultivars with lower cold tolerance were more sensitive to exogenous BRs, exhibiting a stronger response [12].

Abscisic acid (ABA) is another key hormone in plants that broadly regulates growth, development, and responses to environmental stresses [13]. Yao et al. (2019) found that exogenous application of S-ABA significantly increased endogenous ABA levels in maize leaves, promoting enhanced antioxidant enzyme activity, and thereby mitigating oxidative damage under drought stress, improving maize drought adaptation [14]. Tian et al. (2018) further demonstrated that under cold stress, an appropriate concentration of exogenous ABA increased maize root biomass, enhanced PAL and PPO enzyme activities, and significantly elevated the total phenol and flavonoid content, thereby exhibiting effective mitigation of cold stress. Additionally, ABA treatment markedly enhanced the antioxidant capacity of maize seedlings, particularly improving FRAP and ABTS·^+^ scavenging abilities, indicating that exogenous ABA can alleviate the adverse effects of low temperature on maize [15].

Plant hormones interact through intricate signal transduction pathways to jointly regulate the physiological and metabolic processes of plants. In this study, cold-tolerant maize variety LH82 and cold-sensitive variety Shen137 were selected to investigate the response of maize to low-temperature stress following the exogenous application of brassinosteroids (BR) and abscisic acid (ABA), both individually and in combination. By assessing physiological and biochemical indices during the seedling stage and integrating RNA-seq analysis, this study aimed to elucidate the interaction between BR and ABA under low-temperature stress and its role in regulating maize cold tolerance. The findings provide new theoretical support for employing plant hormones to enhance maize cold tolerance and offer valuable insights into the rational regulation of hormones in practical agricultural applications.

## 2. Results

### 2.1. Effects of Exogenous BR and ABA on Maize Seedling Growth Under Cold Stress

**Figure 1:**
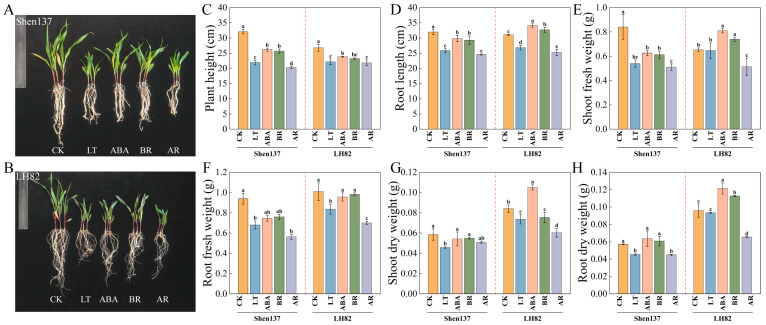
Seedling phenotypes under different treatments. (**A**,**B**) Effects of exogenous hormones on maize seedling morphology under cold stress. (**C**) Root length. (**D**) Seedling height. (**E**) Aboveground fresh weight. (**F**) Belowground fresh weight. (**G**) Aboveground dry weight. (**H**) Belowground dry weight. Different lowercase letters indicated that there were significant differences between treatments by the Duncan test (*p* < 0.05).

To investigate the effects of cold stress and exogenous hormones on maize seedling growth and development, this study treated SHEN 137 (cold-sensitive) and LH82 (cold-tolerant) seedlings with 0.1 mg/L BR, 5 mg/L ABA, and a combined solution (AR) of 5 mg/L ABA plus 0.1 mg/L BR. Significant morphological differences were observed between the two inbred lines under the various treatments (Figure 1A,B). Cold stress (LT) significantly inhibited the growth parameters of both SHEN 137 and LH82, with the cold-sensitive variety SHEN 137 being more adversely affected. Exogenous application of either BR or ABA significantly alleviated the growth inhibition induced by cold stress (Figure 1C–H). BR treatment increased the aboveground and belowground dry weights of SHEN 137 by 18.49% and 39.91%, respectively, whereas LH82 exhibited more pronounced increases of 49.34% and 30.05%, respectively. ABA treatment showed a similar trend, although the increases were slightly lower compared to those observed with BR treatment. However, the AR treatment did not demonstrate a synergistic effect; rather, it exacerbated growth inhibition in certain parameters. For instance, the belowground dry weight of LH82 decreased by 29.84% relative to the cold stress (LT) treatment, suggesting potential interactions between the two hormones.

### 2.2. Effects of Exogenous BR and ABA on the Physicochemical Properties of Maize Seedlings Under Cold Stress

**Figure 2:**
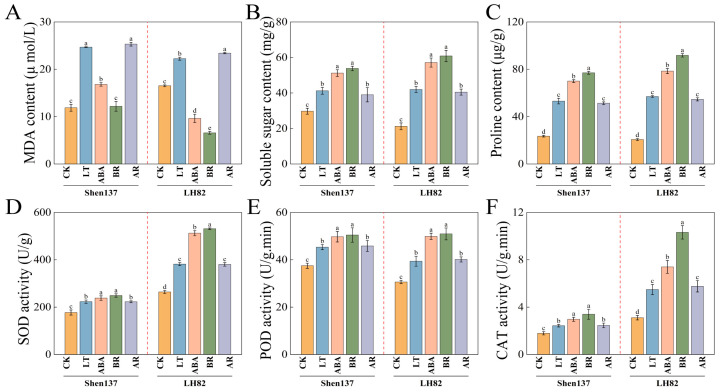
Physiological changes under different treatments. (**A**) MDA content. (**B**) Proline content. (**C**) Soluble sugar content. (**D**) SOD activity. (**E**) POD activity. (**F**) CAT activity. Different lowercase letters indicate significant difference.

Cold stress (LT) significantly increased the levels of malondialdehyde (MDA), proline (Pro), soluble sugars, and the activities of antioxidant enzymes, including superoxide dismutase (SOD), peroxidase (POD), and catalase (CAT). The elevated MDA levels indicate that the plants suffered severe oxidative damage (Figure 2). Exogenous brassinosteroid (BR) treatment significantly enhanced the antioxidant capacity of the plants, particularly in the LH82 variety, where CAT activity increased by 87.92% and MDA levels decreased by 70.39%. Abscisic acid (ABA) treatment similarly increased antioxidant enzyme activities, though the increases were slightly lower than those observed with BR treatment. Furthermore, both BR and ABA treatments significantly increased the contents of proline and soluble sugars, which help maintain cellular osmotic balance. However, the AR treatment did not significantly improve these indices; in some cases (e.g., the MDA level in LH82) it even worsened slightly, increasing by 5.25%. These results further support the possibility of an interaction between BR and ABA under cold stress.

### 2.3. RNA-seq Data Quality Inspection and Sequencing Results

To ensure the robustness and reliability of the RNA-seq data, RNA-seq analysis was performed on leaf samples from the cold-sensitive inbred line Shen137 at the three-leaf stage (V3). A total of 45 leaf samples were collected, with three biological replicates for each treatment condition. Each biological replicate consisted of pooled tissues from three individual plants to account for biological variability. The total RNA concentration and quality of these samples were assessed, and the results indicated that the RNA purity and integrity of all samples met the detection standards (Table S1), fulfilling the requirements for sequencing. After quality control of the raw data, a total of 100.21 Gb of clean data was obtained. The Q30 base percentage for all samples was ≥95.21%, and the GC content ranged from 51.77% to 53.75%. The clean data were then mapped to the maize reference genome (Zm_B73_REFERENCE_NAM_5.0.new). The mapping efficiency of clean reads to the reference genome ranged from 86.09% to 89.53% across the 15 samples (Table S2).

### 2.4. Screening and Statistical Analysis of DEGs

**Figure 3:**
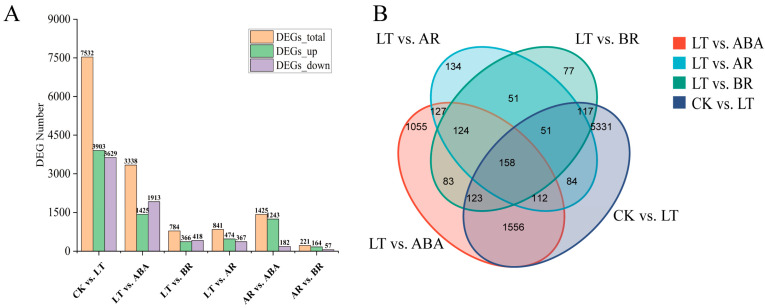
(**A**) The number distribution of up–down DEGs among different comparison groups. (**B**) Venn diagram analysis of DEGs between different comparison groups.

To investigate maize cold tolerance and the response mechanisms to exogenous BR and ABA under cold stress, differential gene expression analysis was performed using DESeq2 (screening criteria: Fold Change ≥ 2, *p* < 0.05). The results showed that cold treatment (LT) led to the identification of 7532 differentially expressed genes (DEGs) in Shen137, with 3903 upregulated and 3629 downregulated (Figure 3A). Under cold stress, the application of exogenous hormones induced differential gene expression: ABA treatment resulted in 3338 DEGs (1425 upregulated/1913 downregulated), BR treatment led to 784 DEGs (366 upregulated/418 downregulated), and AR treatment detected 841 DEGs (474 upregulated/367 downregulated). Venn diagram analysis revealed that 158 DEGs were shared among the three treatments, while 1055, 77, and 134 DEGs were uniquely expressed under ABA, BR, and AR treatments, respectively (Figure 3B). In summary, exogenous ABA enhances cold stress response by regulating a large number of DEGs, whereas BR may exert its effects through a limited number of key genes.

### 2.5. GO Enrichment Analysis

**Figure 4:**
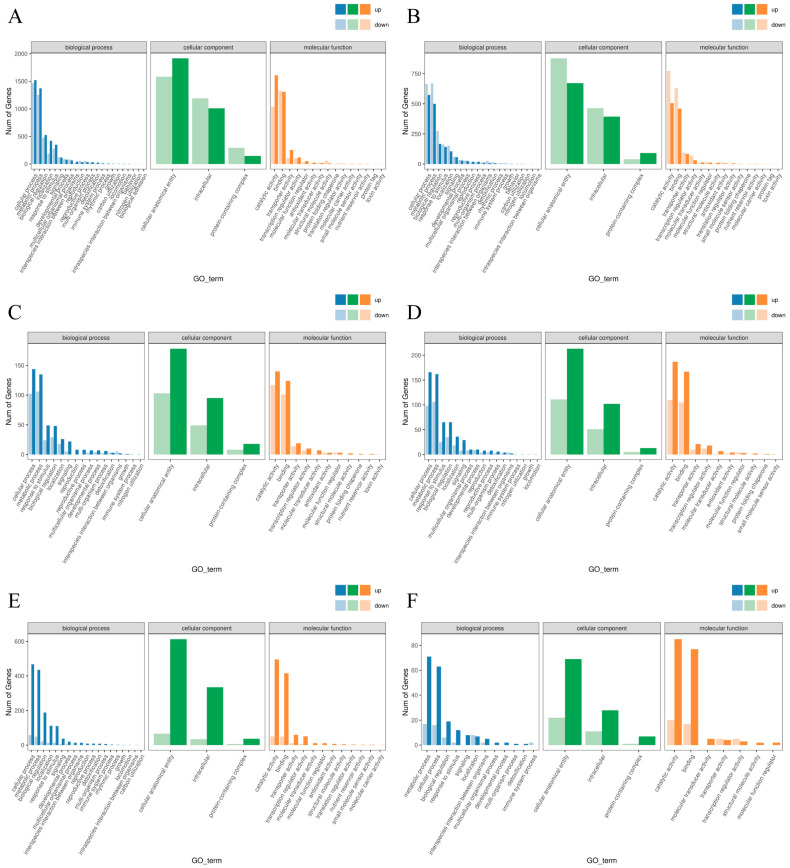
GO analysis of Shen137 DEGs under cold stress and hormone treatment. (**A**) CK vs. LT. (**B**) LT vs. ABA. (**C**) LT vs. BR. (**D**) LT vs. AR. (**E**) ABA vs. AR. (**F**) BR vs. AR.

GO functional enrichment analysis of differentially expressed genes (DEGs) under various treatments was performed to identify their key biological functions. Compared to normal temperature conditions, DEGs in Shen137 under cold stress were primarily enriched in biological processes (BPs) such as cellular processes, metabolic processes, biological regulation, localization, response to stimuli, signaling, and development. The major enriched cellular components (CCs) included cellular anatomical entities, intracellular complexes, and protein-containing complexes. The key enriched molecular functions (MFs) involved catalytic activity, binding activity, transporter activity, and transcription regulator activity (Figure 4A). Under cold stress, the major enriched categories of annotated genes in Shen137 following exogenous ABA, BR, and AR treatments were largely consistent with those identified under cold stress alone. However, within the same enrichment categories, ABA treatment resulted in fewer upregulated genes and more downregulated genes (Figure 4B), whereas BR treatment exhibited the opposite trend, with more upregulated genes and fewer downregulated genes (Figure 4C). This suggests that exogenous ABA and BR exert distinct regulatory effects on gene expression. Exogenous ABA may enhance cold stress tolerance primarily by downregulating genes involved in cellular and metabolic processes, thereby reducing cellular metabolic rates and conserving energy. In contrast, BR may improve cold tolerance mainly by upregulating specific genes. Furthermore, compared to individual ABA and BR treatments, AR treatment resulted in a substantially higher number of upregulated genes across all enriched categories, while the total number of DEGs in the ABA treatment was significantly greater than that in the BR treatment (Figure 4E,F). This indicates an interaction between the two hormones in regulating gene expression. The combined application of ABA and BR may trigger a distinct gene expression pattern compared to individual treatments, activating novel signaling pathways or metabolic processes and leading to widespread changes in gene expression. Additionally, under cold stress, exogenous ABA may play a more dominant role in regulating gene expression.

### 2.6. KEGG Enrichment Analysis

**Figure 5:**
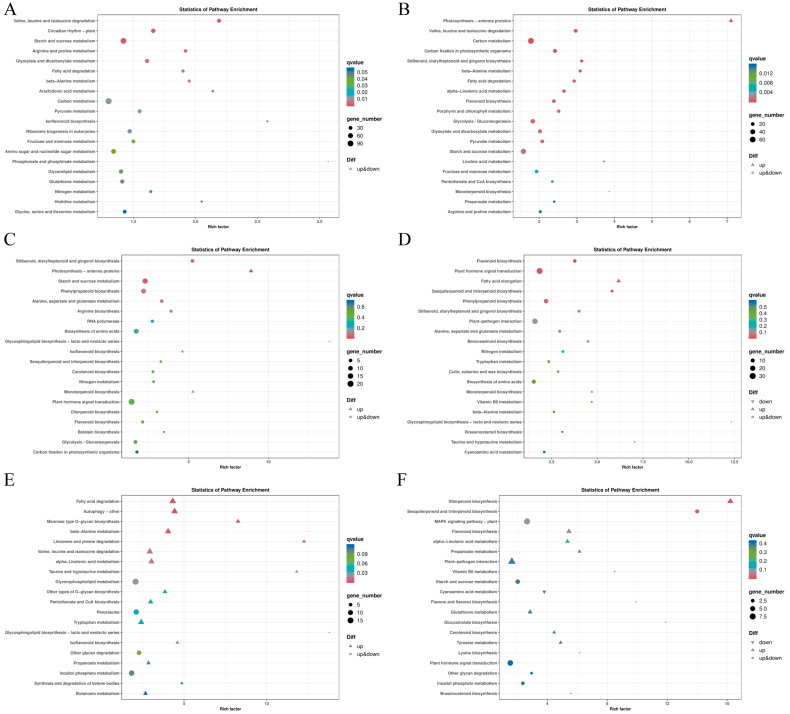
KEGG scatter plot of Shen 137 DEG under cold stress and hormone treatment. The horizontal axis represents the ratio of the number of differentially expressed genes annotated to the KEGG pathway to the total number of differentially expressed genes, and the vertical axis represents the KEGG pathway to the total number of differentially expressed genes, representing the KEGG pathway. The size of the points represents the number of genes annotated to the KEGG pathway. The color ranges from red to purple, representing the significance of the enrichment. (**A**) CK vs. LT. (**B**) LT vs. ABA. (**C**) LT vs. BR. (**D**) LT vs. AR. (**E**) ABA vs. AR. (**F**) BR vs. AR.

To further investigate the functions of DEGs, KEGG functional annotation was performed for DEGs in different treatment groups. The top 20 pathways with the lowest q-values were selected for KEGG enrichment analysis. In the CK vs. LT comparison, DEGs were primarily enriched in metabolic pathways such as starch and sucrose metabolism, circadian rhythm (plant), glyoxylate and dicarboxylate metabolism, valine, leucine, and isoleucine degradation, arginine and proline metabolism, fatty acid degradation, and beta-alanine metabolism. These enriched pathways may be directly related to the response of Shen137 to cold stress (Figure 5A). In the LT vs. ABA comparison, DEGs were significantly enriched in metabolic pathways including photosynthesis–antenna proteins, carbon metabolism, carbon fixation in photosynthetic organisms, valine, leucine, and isoleucine degradation, stilbenoid, diarylheptanoid, and gingerol biosynthesis, as well as beta-alanine metabolism (Figure 5B). These results suggest that exogenous ABA may enhance cold tolerance by regulating gene expression in these metabolic pathways, improving photosynthetic efficiency, modulating energy metabolism, and promoting the synthesis of antioxidant compounds. In the photosynthesis–antenna protein pathway, exogenous ABA may enhance photosynthetic efficiency by upregulating the expression of chlorophyll a/b-binding proteins in light-harvesting complex I (LHCA1, LHCA2, and LHCA4) and light-harvesting complex II (LHCB1, LHCB2, LHCB3, LHCB4, LHCB5, and LHCB6). This enhancement may provide greater energy support, enabling plants to better adapt to cold stress (Table S3). In the LT vs. BR comparison, DEGs were primarily enriched in pathways such as photosynthesis–antenna proteins, stilbenoid, diarylheptanoid, and gingerol biosynthesis, starch and sucrose metabolism, phenylpropanoid biosynthesis, alanine, aspartate, and glutamate metabolism, and arginine biosynthesis (Figure 5C). The enrichment of these pathways suggests that BR may enhance cold tolerance in maize by optimizing photosynthesis and antioxidant responses. In the phenylpropanoid biosynthesis pathway, exogenous BR primarily regulates the differential expression of genes such as phenylalanine/tyrosine ammonia-lyase, 4-coumarate-CoA ligase, cinnamoyl-CoA reductase, and shikimate O-hydroxycinnamoyl transferase. This regulation may enhance lignin biosynthesis, antioxidant responses, and cell wall reinforcement, thereby improving maize adaptation to cold stress (Table S4). In the LT vs. AR comparison, DEGs were mainly enriched in pathways such as plant hormone signal transduction, fatty acid elongation, phenylpropanoid biosynthesis, flavonoid biosynthesis, sesquiterpenoid and triterpenoid biosynthesis, and stilbenoid, diarylheptanoid, and gingerol biosynthesis (Figure 5D). In the ABA vs. AR comparison, DEGs were significantly enriched in pathways such as autophagy-other, fatty acid degradation, valine, leucine, and isoleucine degradation, beta-alanine metabolism, mannose-type O-glycan biosynthesis, and limonene and pinene degradation (Figure 5E). In the BR vs. AR comparison, DEGs were mainly enriched in pathways including diterpenoid biosynthesis, sesquiterpenoid and triterpenoid biosynthesis, flavonoid biosynthesis, and the MAPK signaling pathway in plants (Figure 5F).

### 2.7. RT-qPCR Verification

**Figure 6:**
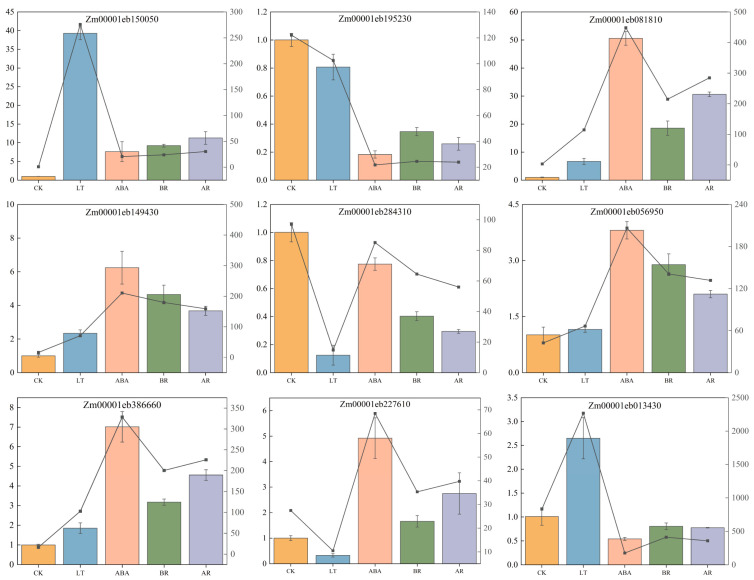
RT-qPCR was performed to validate the expression of nine randomly selected genes. ACTIN was used as the internal reference to normalize mRNA expression levels (mean of three biological replicates). The validation results of transcriptome data were presented using bar charts (RT-qPCR) and line graphs (RNA-Seq) simultaneously.

To further validate the reliability of the transcriptome sequencing results, this study randomly selected nine common differentially expressed genes (DEGs) under low-temperature stress for quantitative real-time PCR (qRT-PCR) verification (Figure 6). The relative expression levels of these nine genes exhibited consistent trends with the RNA-seq sequencing results, which fully confirmed the reliability of the sequencing data.

### 2.8. Weighted Gene Co-Expression Network Analysis (WGCNA) Analysis

#### 2.8.1. Construction of Gene Co-Expression Module

**Figure 7:**
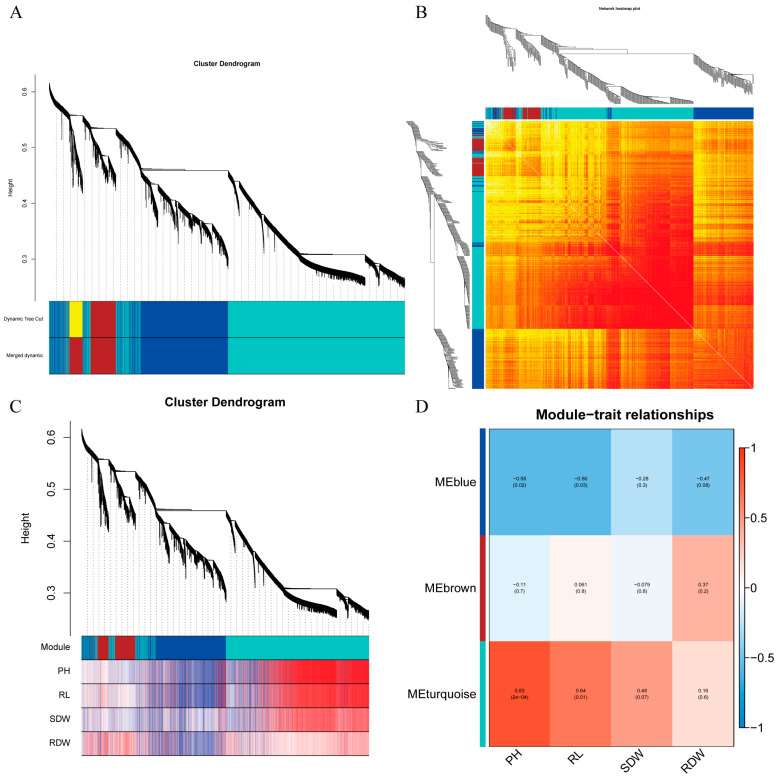
Module construction based on WGCNA. (**A**) Gene network modules. (**B**) Heatmap of the gene co-expression network. (**C**) Phylogenetic tree of genes and heatmap of trait correlations. (**D**) Heatmap of correlations between modules and traits. The closer the correlation is to an absolute value of 1, the stronger the association between the trait and the genes within the module.

To investigate the relationship between gene expression in RNA-seq data and cold tolerance phenotypic traits in maize, this study employed weighted gene co-expression network analysis (WGCNA) to construct a gene co-expression network and identified three co-expression modules associated with phenotypic traits (Figure 7A,B). Module analysis revealed that plant height, root length, and aboveground dry weight were significantly positively correlated with the turquoise module, while belowground dry weight exhibited a significant correlation with the brown module (Figure 7C,D).

#### 2.8.2. Functional Analysis of Gene Module

**Figure 8:**
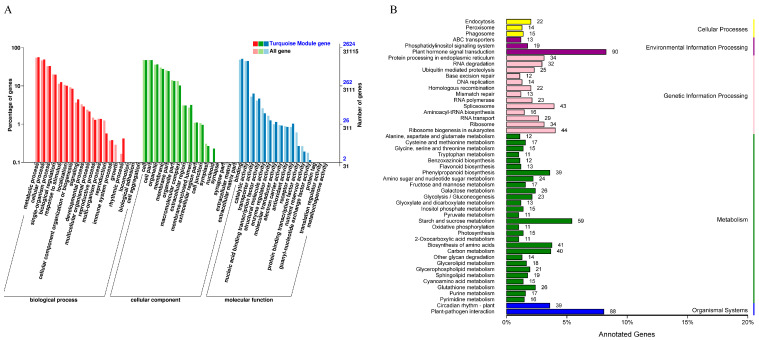
Functional analysis of genes in the turquoise module. (**A**) GO enrichment analysis. (**B**) KEGG enrichment analysis.

To further investigate the functions of genes associated with cold tolerance, we performed GO and KEGG analyses on genes within the turquoise module, which exhibited the highest correlation with cold tolerance phenotypic data (Figure 8). GO enrichment analysis revealed that genes in this module were primarily associated with biological processes (BP) such as metabolic processes, cellular processes, single-organism processes, biological regulation, and responses to stimuli. The enriched cellular components (CC) included the cell, cell part, membrane, and membrane part. The enriched molecular functions (MF) comprised binding activity, catalytic activity, transporter activity, and nucleic acid binding transcription factor activity. KEGG pathway analysis indicated that the key enriched pathways included plant hormone signal transduction, plant–pathogen interaction, starch and sucrose metabolism, ribosome biogenesis in eukaryotes, spliceosome, and amino acid biosynthesis.

#### 2.8.3. Visualization Analysis of Core Genes of the Gene Module

**Figure 9:**
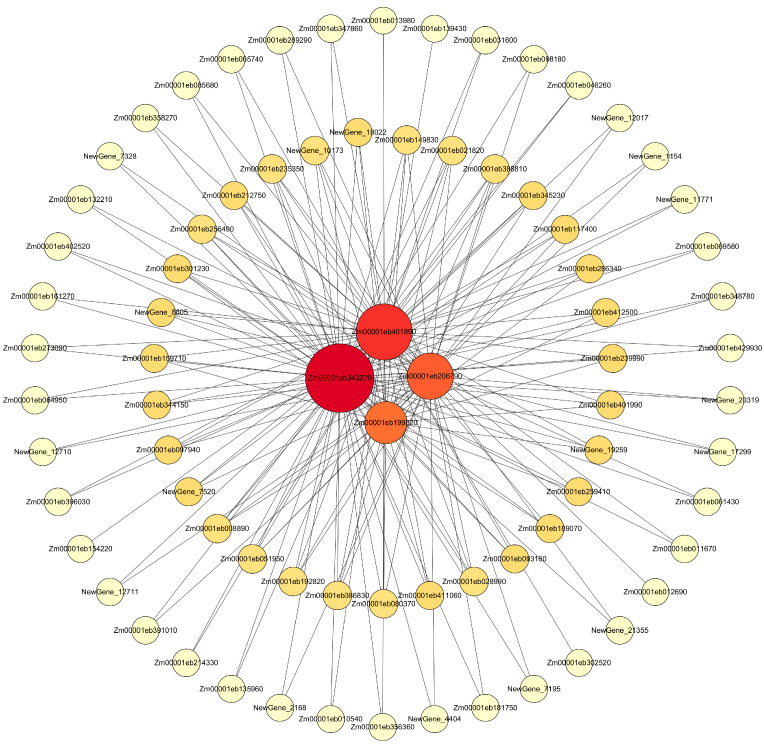
Interaction network analysis of hub genes in the turquoise module. The size and color of the nodes represent the level of gene connectivity.

A co-expression network of the turquoise module (top 150 weighted genes) was constructed, and genes with high connectivity were selected as core genes: Zm00001eb343270, Zm00001eb401890, Zm00001eb206790, and Zm00001eb199820 (Figure 9). The gene annotations were as follows: ubiquitin-conjugating enzyme E2 O, tubulin–tyrosine ligase-like protein 12, systemic acquired resistance negative regulator SNI1, and nucleolar phosphoprotein.

## 3. Discussion

### 3.1. Effects of Low Temperature and Exogenous ABA and BRs on Maize Seedling Growth

Low temperature is one of the major environmental stresses that adversely affects plant growth, development, and geographical distribution [16]. In recent years, the increasing frequency of extreme weather events has significantly impacted crop growth. To cope with the adverse effects of low temperatures, plants have evolved complex mechanisms to withstand cold stress. Among these, the plant hormone system is considered a crucial strategy for cold stress resistance [17]. In this study, low-temperature treatment significantly reduced maize seedling growth parameters, including plant height, root length, fresh weight, and dry weight. The application of exogenous ABA or BR alleviated cold-induced damage and enhanced cold tolerance. However, under low-temperature stress, the simultaneous application of BR and ABA did not improve maize seedling growth or enhance cold tolerance, suggesting a potential interaction between BR and ABA in maize seedlings. Physiological indicators of maize seedlings under different treatments were measured in this study. The results showed that malondialdehyde (MDA), proline, soluble sugar content, and antioxidant enzyme activity were significantly increased under low-temperature stress. The application of exogenous ABA or BR at optimal concentrations enhanced maize cold tolerance by reducing MDA content, increasing proline and soluble sugar levels, and improving antioxidant enzyme activity. However, under low-temperature stress, the physiological indicators of maize seedlings treated with both BR and ABA showed no significant changes. These findings suggest that exogenous ABA and BR enhance maize cold tolerance by reducing cellular oxidative damage, improving osmotic regulation, and maintaining water balance and stability, thereby protecting cells from oxidative stress. However, the two hormones may interfere with each other under cold stress, affecting their respective signaling pathways or mechanisms of action. This further indicates that BR and ABA may interact in maize seedlings under low-temperature stress.

### 3.2. Mechanism of Exogenous ABA in Enhancing Cold Tolerance in Maize Seedlings

Abscisic acid (ABA) plays a crucial role in regulating plant growth, development, and stress adaptation. Li et al. have demonstrated that ABA can enhance cold stress tolerance in winter wheat by increasing antioxidant enzyme activity [18]. Transcriptomic analysis revealed that under cold stress, treatment of Shen137 maize with exogenous ABA resulted in the identification of 3338 differentially expressed genes (DEGs), of which 57.31% were downregulated. This suggests that exogenous ABA may enhance maize cold tolerance by downregulating genes involved in metabolic pathways or physiological processes that are detrimental to cold tolerance. Among the DEGs, the genes encoding PP2C (protein phosphatase 2C) and JAZ (jasmonate ZIM-domain) proteins were downregulated in Shen137. Studies have shown that PP2C acts as a negative regulator of the ABA signaling pathway [19], while JAZ proteins serve as negative regulators of the jasmonic acid (JA) signaling pathway [20]. ABA and JA exhibit synergistic effects [21]. ABA increases JA levels by promoting the expression of the MYC2 gene in the JA biosynthetic pathway [22], thereby enhancing plant cold tolerance. In Shen137, the coordinated downregulation of JAZ protein genes together with the expression of the transcription factor MYC2 may promote the JA signaling pathway, thus improving maize cold tolerance. Additionally, the downregulation of PP2C gene expression enhances SnRK2 activity. SnRK2 can phosphorylate downstream transcription factors such as ABF, thereby modulating ABA responses and increasing the cold resistance of maize seedlings.

Studies have found that auxin homeostasis is critical for the normal growth of crops under cold stress [23]. The upregulation of AUX1 facilitates auxin influx, while the upregulation of ARF, downregulation of GH3, and coordinated changes in AUX/IAA collectively modulate the auxin response. These changes may help regulate the growth and development of maize seedlings under cold stress, thereby enhancing their adaptability to low-temperature environments. Furthermore, shikimate O-hydroxycinnamoyl transferase may influence cellular antioxidant capacity by modulating flavonoid content [24]. In Shen137, the upregulated expression of the shikimate O-hydroxycinnamoyl transferase gene may enhance maize cold tolerance by increasing flavonoid biosynthesis, thereby improving the plant’s antioxidant capacity.

Therefore, the activation of the ABA signaling pathway, the synergistic action of the JA signaling pathway, the regulation of auxin homeostasis, and the enhancement of antioxidant metabolism all contribute to the ABA-mediated improvement of cold tolerance.

### 3.3. Mechanism of Exogenous BR in Enhancing Cold Tolerance in Maize Seedlings

Brassinosteroids (BRs) are a class of steroid hormones that regulate cell elongation, division, and differentiation in plants, playing a critical role in plant growth and development [25,26,27]. Furthermore, BRs are also important in mediating responses to stresses such as salinity, drought, and cold. Xia et al. (2009) demonstrated that BRs can significantly enhance plant tolerance to cold stress by inducing the expression of regulatory genes (e.g., RBOH, MAPK1, and MAPK3) and genes involved in defense and antioxidant responses [28]. This conclusion has been further validated in crops such as rice and wheat [29,30].

Physiological studies have shown that BRs can improve the cold tolerance of maize seedlings. Transcriptomic analysis revealed that the genes MKK4_5 (mitogen-activated protein kinase kinase 4/5) and MKK3 (mitogen-activated protein kinase kinase 3) were significantly upregulated in the plant–pathogen interaction pathway. As key regulators in stress signaling networks, MAPKKs enhance plant stress tolerance by modulating downstream defense responses, thereby facilitating efficient signal transduction in response to cold stress [31].

Chlorophyll a/b binding proteins (CAB) are lipoproteins of the photosystem II (PSII) light-harvesting complex in higher plants, essential for photosynthesis and widely involved in the regulation of responses to biotic stress [32,33]. Zhang et al. found that the overexpression of the BnLhcb3.4 gene significantly enhanced the freezing tolerance in transgenic Arabidopsis and increased its sensitivity to abscisic acid (ABA) [34]. In this study, BR treatment upregulated the expression of CAB-related genes (*Zm00001eb207130*, *Zm00001eb161390*, *Zm00001eb161410*, *Zm00001eb343900*, and *Zm00001eb324240*). Previous studies have shown that plants lacking Lhcb6 accumulate higher levels of superoxide and hydrogen peroxide and suffer more severe oxidative damage in the field, suggesting that Lhcb6 may play a role in mitigating oxidative stress and photoprotection under natural conditions [35]. We hypothesize that exogenous BR may slow chlorophyll degradation and enhance the accumulation of antioxidants, thereby improving plant cold tolerance.

Moreover, in the phenylpropanoid biosynthesis pathway, BR regulates the differential expression of genes such as phenylalanine/tyrosine ammonia-lyase, 4-coumarate-CoA ligase, cinnamoyl-CoA reductase, and shikimate O-hydroxycinnamoyl transferase, thereby promoting the synthesis of phenylpropanoid compounds. These compounds possess antioxidant properties that enable them to scavenge reactive oxygen species generated under cold stress, thereby alleviating oxidative damage [36]. Additionally, in the stilbenoid, diarylheptanoid, and gingerol biosynthesis pathways, BR may regulate the expression of genes such as trans-resveratrol di-O-methyltransferase and caffeoyl-CoA O-methyltransferase, leading to the synthesis of compounds with antioxidant and cold-protective properties.

In summary, BR enhances the adaptation of maize seedlings to cold stress by integrating MAPK signaling cascades, reinforcing the photoprotective system, and synergistically modulating the antioxidant metabolic network.

### 3.4. Regulation of Maize Cold Tolerance by the Interaction Between Exogenous ABA and BR

Interactions among plant hormones significantly influence stress tolerance. In this study, we found that exogenous ABA or BR applied individually alleviated cold-induced damage by enhancing antioxidant capacity and osmotic adjustment; however, their combined application (AR treatment) failed to produce a synergistic effect and instead exacerbated certain physiological injuries (Figure 1C–H and Figure 2A–F).

Transcriptomic analysis revealed that AR treatment affected hormone signaling in Shen137 as follows: in the IAA signaling pathway, the expression of AUX1, AUX/IAA, and ARF genes was upregulated; in the ABA signaling pathway, SnRK2 gene expression was upregulated while ABF gene expression was downregulated; in the GA signaling pathway, DELLA protein expression exhibited both up- and downregulation, with PIF3 gene expression being upregulated; in the BR signaling pathway, the expressions of BAK1 and BRI1 were variably regulated, and TCH4 gene expression was downregulated; in the JA signaling pathway, MYC2 gene expression was upregulated; and in the SA signaling pathway, TGA gene expression was downregulated while PR1 gene expression was upregulated.

As a central hormone in cold resistance, ABA’s key negative regulator, PP2C (*Zm00001eb098220*, *Zm00001eb107130*, and *Zm00001eb309920*), was upregulated in the ABA vs. AR comparison, thereby inhibiting SnRK2 kinase activity [37] and leading to downregulation of the downstream ABA-responsive transcription factor ABF [38]. Meanwhile, BIN2 kinase, a negative regulator in the BR signaling pathway [39] and an interacting partner of SnRK2.2, mediates the phosphorylation of SnRK2 [40]. Moreover, the BR signaling receptors BAK1/BRI1 (*Zm00001eb343100*) were significantly activated in AR treatment, which may further impede ABA signal transduction, leading to impaired maize antioxidant activity and osmotic regulation, ultimately reducing cold tolerance in maize seedlings.

Under AR treatment, key genes in the auxin signaling pathway—including AUX1 (*Zm00001eb280540*), ARF (*Zm00001eb170210*), and SAUR (*Zm00001eb050780*)—were significantly upregulated. Additionally, DELLA proteins in the GA signaling pathway regulate the stability of PIF proteins. In this study, the downregulation of the DELLA gene (*Zm00001eb325750*) positively influenced PIF3 activity. The core transcription factor BZR1 in the BR signaling pathway may form a BAP module (BZR1-ARF-PIF3) with ARF and PIF, synergistically driving the expression of growth-related genes [41,42]. Furthermore, the expression of the core transcription factor MYC2 (*Zm00001eb250670*) in the jasmonic acid (JA) pathway was enhanced, potentially due to JAZ protein degradation (with JAZ1/3 downregulated in the BR vs. AR group), thereby relieving inhibition on MYC2 and forming a cascade effect characterized as “JA enhancement—ABA suppression” [43].

Co-expression network analysis identified the core gene SNI1 (*Zm00001eb206790*), which acts as a negative regulator of systemic acquired resistance (SAR) [44]. Additionally, ubiquitin-conjugating enzyme E2 (*Zm00001eb343270*) plays a key role in ubiquitination by catalyzing ubiquitin transfer [45]. In the salicylic acid (SA) signaling pathway, TGA transcription factor expression was downregulated, whereas PR1 gene expression was significantly upregulated. This phenomenon may be attributed to SNI1-mediated ubiquitination and degradation of the SA signaling core factor NPR1, resulting in inhibited TGA activity [46]. Concurrently, the upregulation of PR1 may result from the synergistic activation via the interaction between WRKY transcription factors and the core transcription factor MYC2 in the JA pathway [47,48]. In Arabidopsis, overexpression of NtWRKY65 enhanced salt tolerance in transgenic plants, with its cis-regulatory elements responsive to IAA, ABA, and SA [49]. Under cold stress, WRKY33 directly targets and induces various kinases, transcription factors, and molecular chaperone genes (e.g., CDPK11, MYBS3, and BAG6), thereby enhancing plant cold tolerance [50]. In this study, exogenous AR treatment resulted in the downregulation of WRKY33 (Zm00001eb337270), which may lead to increased H_2_O_2_ accumulation and decreased CAT and SOD activities, ultimately reducing the cold tolerance of maize seedlings.

Notably, AR treatment significantly upregulated autophagy-related genes (ATG1/3/4/7/8/13) and fatty acid β-oxidation genes (e.g., acyl-CoA oxidase). We hypothesize that ABA and BR signaling may activate autophagy via ROS and BZR1 [51,52], while excessive activation of fatty acid β-oxidation leads to an imbalance in acetyl-CoA supply, further exacerbating mitochondrial ROS accumulation [53]. Additionally, the downregulation of WRKY33 (*Zm00001eb337270*) may suppress CAT/SOD expression [54], reducing H_2_O_2_ scavenging capacity and establishing a vicious cycle of oxidative stress.

**Figure 10:**
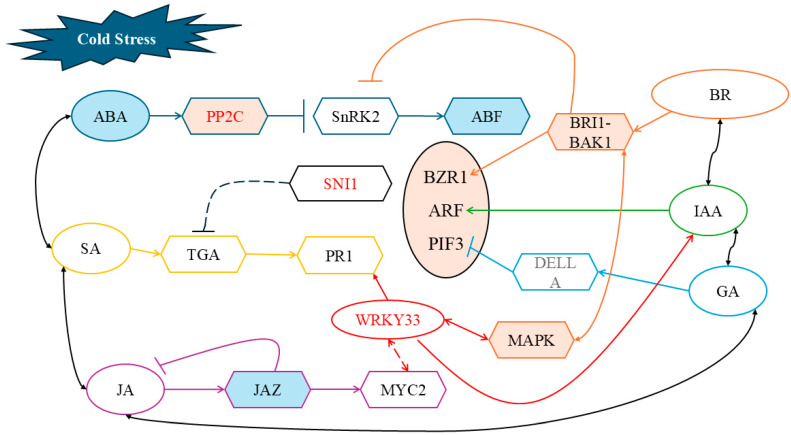
A model of the interaction between exogenous abscisic acid (ABA) and brassinosteroid (BR) in response to cold stress in maize. Blue fill indicates exogenous ABA-responsive nodes, orange fill indicates exogenous BR-responsive nodes, and red font indicates nodes responsive to combined exogenous ABA-BR treatment. Blue lines represent ABA regulation, red lines represent potential WRKY33 regulation, and dashed lines indicate hypothetical regulatory relationships.

In summary, the interaction between ABA and BR occurs at multiple plant hormone signaling nodes. The cold tolerance is weakened through the growth deviation of the BAP module, the signal conflict between SA and JA, as well as the imbalance of autophagy and energy metabolism (Figure 10).

## 4. Materials and Methods

### 4.1. Experimental Materials

The cold-sensitive maize inbred line Shen137 and the cold-tolerant maize inbred line LH82 used in this study were provided by the Maize Research Group at Gansu Agricultural University. Exogenous abscisic acid (ABA, C_15_H_20_O_4_) was purchased from Shanghai YuanYe Biotechnology Co., Ltd. (Shanghai, China), and brassinosteroid (BR, C_28_H_48_O_6_) was purchased from Solarbio (Beijing, China).

### 4.2. Experimental Design and Treatment

Based on previously published experimental results [55] from our laboratory, the optimal hormone concentrations were determined, and five treatments were applied to two genotypes. The experimental treatments were as follows: (1) CK, normal temperature (25 ± 1 °C) + distilled water; (2) LT, low temperature (10 ± 1 °C) + distilled water; (3) ABA, low temperature (10 ± 1 °C) + 5 mg/L ABA; (4) BR, low temperature (10 ± 1 °C) + 0.1 mg/L BR; (5) AR, low temperature (10 ± 1 °C) + 5 mg/L ABA + 0.1 mg/L BR.

Sterilized perlite was uniformly mixed with sterile water at a ratio of 5 g:1 mL. Ten soaked seeds were then selected and sown into nutrient pots containing sterilized perlite. Subsequently, the pots were placed in a climate chamber set at 25 ± 1 °C, 60–80% relative humidity, a light intensity of 600 μmol/(s·m^2^), and a photoperiod of 12 h light/12 h dark, and grown until the three-leaf stage. They were then directly transferred to another climate chamber set at 10 ± 1 °C, 60–80% relative humidity, a light intensity of 600 μmol/(s·m^2^), and a photoperiod of 12 h light/12 h dark for cold treatment for 7 days. Seedlings grown at 25 ± 1 °C with sterile water treatment served as the positive control (CK), whereas those grown at 10 ± 1 °C served as the negative control (LT). Every two days at 9:00 AM, 50 mL of the treatment solution was applied (25 mL via foliar spray and 25 mL applied to the roots). Each treatment was performed with three independent physical replicates, resulting in a total of 60 leaf samples.

### 4.3. Index Measurement

#### 4.3.1. Growth Index Measurement

After washing off the perlite from the seedling roots, the surface moisture was removed using filter paper. The seedling and root lengths of maize were measured with a ruler. The aboveground fresh weight, root fresh weight, aboveground dry weight, and root dry weight of maize were determined using an analytical balance.

#### 4.3.2. Measurement of Physiological Indexes

Malondialdehyde (MDA) content, proline (Pro) content and soluble protein (SP) content were determined according to the method described by Li et al. [56]. The activity of superoxide dismutase (SOD), peroxidase (POD), and catalase (CAT) were determined according to the method described by Wang et al. [57].

### 4.4. Transcriptomic Analysis

#### 4.4.1. Total RNA Extraction and Illumina Deep Sequencing

Leaf collar tissues from the second leaf of Shen137 seedlings at the V3 stage were collected from 45 individual plants. Each treatment was set with three biological replicates. Each biological replicate consisted of pooled tissues from three individual plants to account for biological variability. The tissues were flash-frozen in liquid nitrogen and stored at −80 °C. Total RNA was extracted using the RNA Prep Pure Plant Kit (Tiangen, Beijing, China) and its concentration and purity were measured with a NanoDrop 2000 (Thermo Fisher Scientific, Wilmington, DE, USA). RNA integrity was assessed using the Agilent Bioanalyzer 2100 system in conjunction with the RNA Nano 6000 kit (Agilent Technologies, CA, USA). Qualified samples were used to construct paired-end 150 bp sequencing libraries on the Illumina NovaSeq platform, and sequencing was carried out by Beijing Bimaike Company (Beijing, China).

#### 4.4.2. Quality Assessment of Sequencing Results

Raw data were processed to remove adapter sequences, low-quality reads, and poly-N sequences, yielding clean data. Clean reads were aligned to the B73 reference genome (Zm_B73_REFERENCE_NAM_5.0) using Hisat2, transcripts were assembled with StringTie, and gene expression levels were quantified using the FPKM method [58,59].

#### 4.4.3. Differential Expression Gene Analysis

Differently expressed genes were screened based on DESeq2 (Fold Change ≥ 2, *p* < 0.05), and GO/KEGG function enrichment analysis was performed by clusterProfiler [60].

#### 4.4.4. Analysis of the Gene Co-Expression Network

Genes with FPKM ≥ 1 were included to construct a weighted gene co-expression network (module similarity threshold of 0.25, minimum module size of 30 genes). Module member genes with kME > 0.7 were selected. Cytoscape 3.10.3 was utilized to visualize the interaction network of core modules, and hub genes were identified based on kME values and connectivity.

#### 4.4.5. Real-Time Fluorescence Quantitative PCR (RT-qPCR) Verification of DEGs

Nine common differentially expressed genes were randomly selected, and specific primers were designed using Primer-BLAST (Table S5). Amplification was performed on the QuantStudio5 system using the SYBR Green Pro Taq HS kit (Code No. AG11701, www.agbio.com.cn, accessed on 31 March 2025), with ACTIN as the internal control. Relative expression levels were calculated using the 2^−ΔΔCT^ method. Reverse transcription (Evo M-MLV kit(Code No. AG11706, www.agbio.com.cn, accessed on 31 March 2025)) and cDNA storage were conducted following standard protocols [61].

### 4.5. Statistics

Statistical analyses were conducted using Microsoft Excel 2016 and SPSS Statistics 25.0 (one-way ANOVA, *p* < 0.05). Figures were generated using Origin 2022. Data are presented as mean ± standard error.

## 5. Conclusions

This study provides insights into the regulatory mechanisms of exogenous abscisic acid (ABA) and brassinosteroids (BR) in enhancing maize cold tolerance. Physiological analyses revealed that both ABA and BR individually improved maize resistance to cold stress by enhancing antioxidant activity and osmotic regulation. However, their combined application (AR treatment) did not confer additional benefits, suggesting a potential antagonistic interaction. Transcriptomic analysis identified key pathways, including plant hormone signal transduction, fatty acid metabolism, and phenylpropanoid biosynthesis, as being involved in ABA and BR responses. WGCNA highlighted critical candidate genes such as Zm00001eb343270, Zm00001eb401890, Zm00001eb206790, and Zm00001eb199820, which may play pivotal roles in maize cold tolerance. These findings contribute to our understanding of hormone interactions under cold stress and offer a foundation for improving maize stress resilience through targeted hormonal regulation.

## Data Availability

The sequencing data of this study were stored in the SRA database of NCBI, and the accession number was PRJNA1111354.

## Supplementary Materials

The following supporting information can be downloaded at: https://www.mdpi.com/article/10.3390/ijms26073326/s1.
